# SHIV-162P3 Infection of Rhesus Macaques Given Maraviroc Gel Vaginally Does Not Involve Resistant Viruses

**DOI:** 10.1371/journal.pone.0028047

**Published:** 2011-12-02

**Authors:** Athe M. N. Tsibris, Urboshi Pal, Allison L. Schure, Ronald S. Veazey, Kevin J. Kunstman, Timothy J. Henrich, P. J. Klasse, Steven M. Wolinsky, Daniel R. Kuritzkes, John P. Moore

**Affiliations:** 1 Massachusetts General Hospital, Boston, Massachusetts, United States of America; 2 Harvard Medical School, Boston, Massachusetts, United States of America; 3 Cornell University, Ithaca, New York, United States of America; 4 Tulane National Primate Research Center, Covington, Louisiana, United States of America; 5 Northwestern University Feinberg School of Medicine, Chicago, Illinois, United States of America; 6 Brigham and Women's Hospital, Boston, Massachusetts, United States of America; 7 Department of Microbiology and Immunology, Weill Medical College of Cornell University, New York, New York, United States of America; University of Pittsburgh, United States of America

## Abstract

Maraviroc (MVC) gels are effective at protecting rhesus macaques from vaginal SHIV transmission, but breakthrough infections can occur. To determine the effects of a vaginal MVC gel on infecting SHIV populations in a macaque model, we analyzed plasma samples from three rhesus macaques that received a MVC vaginal gel (day 0) but became infected after high-dose SHIV-162P3 vaginal challenge. Two infected macaques that received a placebo gel served as controls. The infecting SHIV-162P3 stock had an overall mean genetic distance of 0.294±0.027%; limited entropy changes were noted across the envelope (gp160). No envelope mutations were observed consistently in viruses isolated from infected macaques at days 14–21, the time of first detectable viremia, nor selected at later time points, days 42–70. No statistically significant differences in MVC susceptibilities were observed between the SHIV inoculum (50% inhibitory concentration [IC_50_] 1.87 nM) and virus isolated from the three MVC-treated macaques (MVC IC_50_ 1.18 nM, 1.69 nM, and 1.53 nM, respectively). Highlighter plot analyses suggested that infection was established in each MVC-treated animal by one founder virus genotype. The expected Poisson distribution of pairwise Hamming Distance frequency counts was observed and a phylogenetic analysis did not identify infections with distinct lineages from the challenge stock. These data suggest that breakthrough infections most likely result from incomplete viral inhibition and not the selection of MVC-resistant variants.

## Introduction

Vaginal intercourse is now the most common mode of HIV-1 transmission worldwide [1], [2]. Microbicide gels containing antiretroviral compounds (ARVs) applied vaginally constitute one plausible intervention strategy [3], [4]. Proof-of-concept for this method of prophylaxis has been obtained in animal models using various ARVs, and a tenofovir-based microbicide gel has shown protective efficacy in women [5]–[10]. However, breakthrough infections can occur in animals and humans for one of several reasons, including non-adherence (in humans), the presumed inadequate delivery of the active drug to its site of action, and the presence of viral variants resistant to the ARV. Tenofovir-related resistance mutations were not detected by standard clinical HIV-1 genotype testing on plasma viral isolates from women who became HIV-infected while using a tenofovir vaginal gel [9]. Nonetheless, it remains relevant to understand what selective effects a vaginal microbicide prophylaxis regimen may have on the infecting viral quasispecies because of general concerns about the spread of drug-resistant variants [3], [4], [11], [12]. Naturally occurring CCR5 antagonist-insensitive virus variants have been reported prior to drug challenge [13]–[15].

HIV transmission most commonly involves viruses that use the CCR5 coreceptor for entry into cells [16], [17]. Accordingly, specific inhibitors that bind to CCR5 can prevent infections of rhesus macaques with CCR5-using viruses, such as SHIV-162P3 [5], [6]. Maraviroc (MVC) is the only CCR5 antagonist approved for treatment of HIV-1 infection [18], [19]. A maraviroc (MVC) vaginal microbicide protected macaques in a dose- and time-dependent manner from high-dose SHIV-162P3 vaginal challenge [10]. However, some breakthrough infections did occur even when MVC was applied at high concentrations in the protective range (gel concentrations of 0.6–5.8 mM) [10]. One explanation is that an insufficient amount of MVC was present in the right place at the right time (pharmacological failure), a second is that some viruses present in the challenge virus stock were partially resistant to MVC and were selected for by the gel (resistance failure). We note that another CCR5 inhibitor, PSC-RANTES, was reported to select for a resistant SHIV-162P3 variant when applied vaginally to macaques, although this conclusion has since been questioned [20], [21]. Here, we investigated whether SHIV-162P3 variants infecting macaques in the presence of a MVC vaginal gel have any genetic and phenotypic characteristics indicative of resistant viruses.

## Results

**Figure 1:**
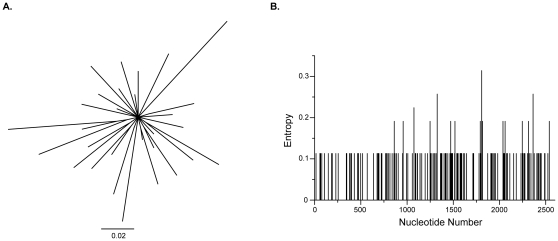
The diversity of the infecting SHIV-162P3 inoculum. (A) A maximum likelihood tree constructed with 42 independent full-length clones isolated from the infecting SHIV-162P3 inoculum. An unrooted tree layout is displayed. The horizontal scale bar represents genetic distance. (B) Entropy plot of inoculum diversity as a function of nucleotide position.

**Figure 2:**
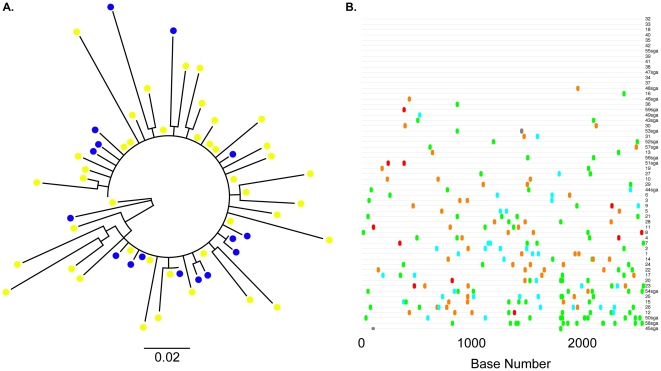
The relationship between SHIV-162P3 stock full-length *env* obtained by standard cloning and single genome amplification. (A) An unrooted maximum likelihood tree constructed with standard clones and 17 previously reported sequences generated by single genome amplification. Blue circles, SGA clones; Yellow circles; standard clones. The horizontal bar represents genetic distance. (B) Highlighter plot indicates the gp160 nucleotide variation between clones. Clones are numbered sequentially; SGA clones are depicted with “sga” after the clone number. Adenine, green; Cytosine, aqua; Thymine, red; Guanine, orange. Grey bars indicate missing sequence.

To characterize the SHIV-162P3 inoculum, we performed a standard clonal analysis of 42 independent full-length *env* clones isolated from the infecting stock. Standard PCR and cloning provided similar measures of population diversity when compared directly to single genome sequencing; sampling bias occurred with either method [22]. A phylogenetic analysis was performed to graphically represent SHIV-162P3 diversity and entropy calculations quantified sequence variation by nucleotide position (**Fig. 1**). Minor sequence differences were present throughout gp160, although several positions in gp120 and gp41 were invariant. An entropy of approximately 0.1 corresponds to one nucleotide difference at a given position in one sequence amongst all 42 SHIV sequences. A diversity estimate of the SHIV-162P3 stock demonstrated an overall mean genetic distance of 0.294±0.027% (standard error), consistent with prior reports [23], [24]. We compared full-length *env* sequences from SHIV-162P3 stock isolated by standard cloning with previously reported sequences generated by single genome amplification (**Fig. 2**) [25]. Sequences obtained by either method generated a SHIV-162P3 consensus sequence that was identical at all nucleotide positions across the length of gp160.

**Figure 3:**
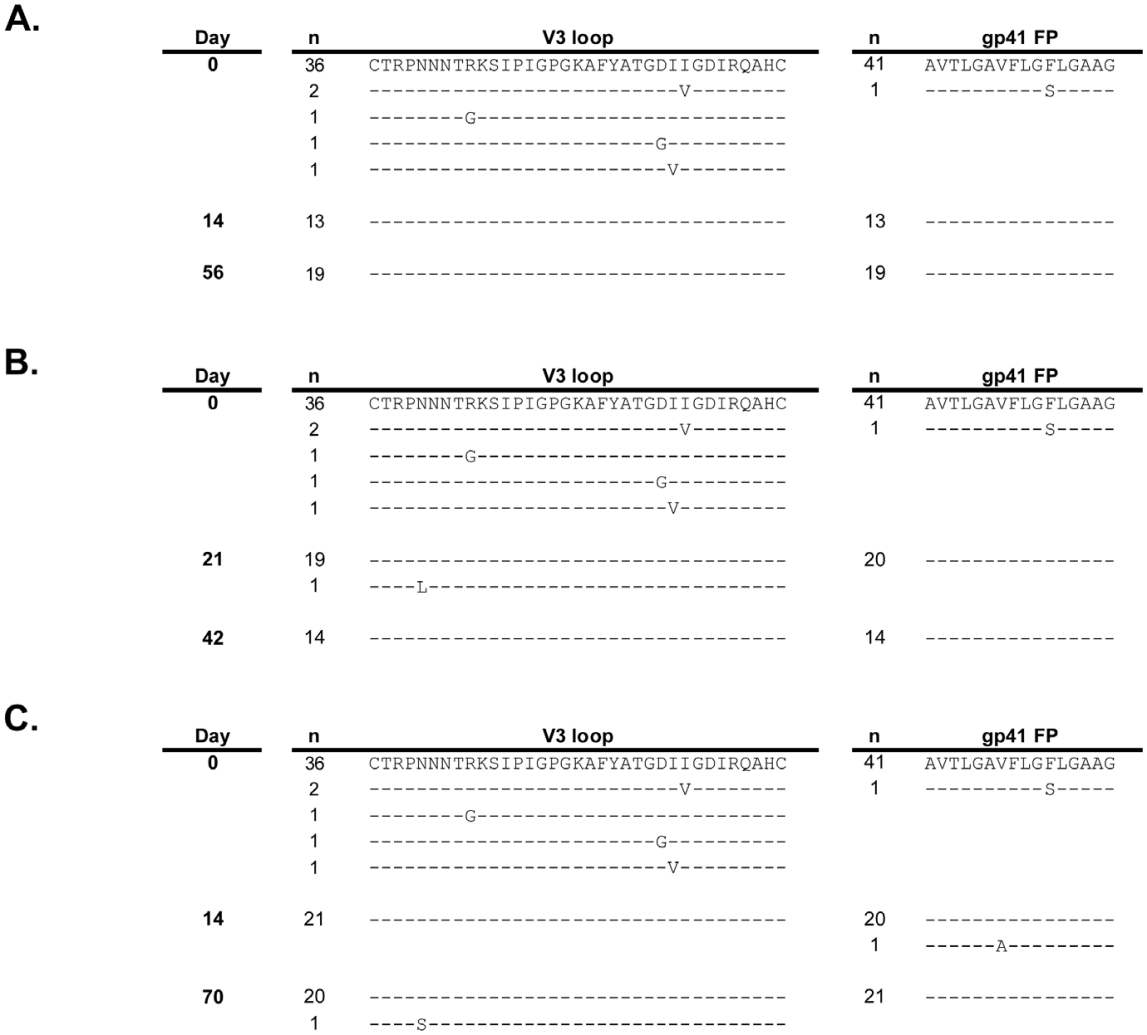
Alignment of gp120 V3 and gp41 fusion peptide sequences obtained pre-challenge and from post-challenge time points 1 and 2. Independent clonal sequences isolated from three MVC-exposed, SHIV-162P3 infected macaques are shown. The pre-challenge sequences are the same for each macaque as they were derived from the SHIV-162P3 challenge stock. Predicted amino acid differences are shown and similarities indicated with dashes. The number of independent clones with the identical sequence is indicated to the left of each sequence. (A) Mac46, (B) Mac73, (C) Mac80. FP, fusion peptide.

To investigate the characteristics of infecting SHIV populations, we studied viral isolates from five rhesus macaques: Mac46, Mac73, and Mac80 received a MVC-containing vaginal gel and macaques CR02 and L375 received a placebo vaginal gel and served as a comparator group. Two time points were assessed for each animal: T_1_, the time of first detectable plasma viremia (day 14 or 21), and T_2_, a later time point (days 56–70). Plasma SHIV RNA levels were quantified over the study duration (**Table 1**). The *env* genotypic changes that occurred in infected macaques were studied by isolating and sequencing multiple independent full-length plasma-derived *env* clones. A clonal full-length *env* sequence analysis demonstrated that, compared to the SHIV-162P3 inoculum, nearly homogeneous wild-type sequence populations were present in the V3 region of gp120 and the gp41 fusion peptide of Mac46, Mac73, and Mac80 (**Fig. 3**). These two envelope regions have been associated with CCR5 inhibitor resistance [26]–[29]. Non-synonymous mutations were identified in T_1_ plasma-derived full-length *env* from all animals; they resulted in a maximum of three amino acid changes per clone, relative to the consensus SHIV162-P3 sequence (data not shown). No consistent amino acid changes in gp160 sequence outside V3 or the gp41 fusion peptide were noted in any of the five infected macaques.

**Table 1:** SHIV Plasma Viral Loads in MVC-treated and comparator macaques.

		SHIV RNA copies/mL on Day:						
Animal	Gel Treatment	0	7	14	21	42	56	70
**CR02**	Control	-	<125	4,616,200	191,166	56,905	221	<125
**L375**	Control	<125	<125	2,357,700	94,787	10,540	-	3,809
**Mac46**	5.8 mM MVC	<165	<165	1,861,200	2,875,900	22,205	25,657	5,443
**Mac73**	2.9 mM MVC	-	<165	<165	464,078	5,691	349	3,995
**Mac80**	0.6 mm MVC	-	<165	80,116	5,609,100	143,598	19,980	3,739

**Figure 4:**
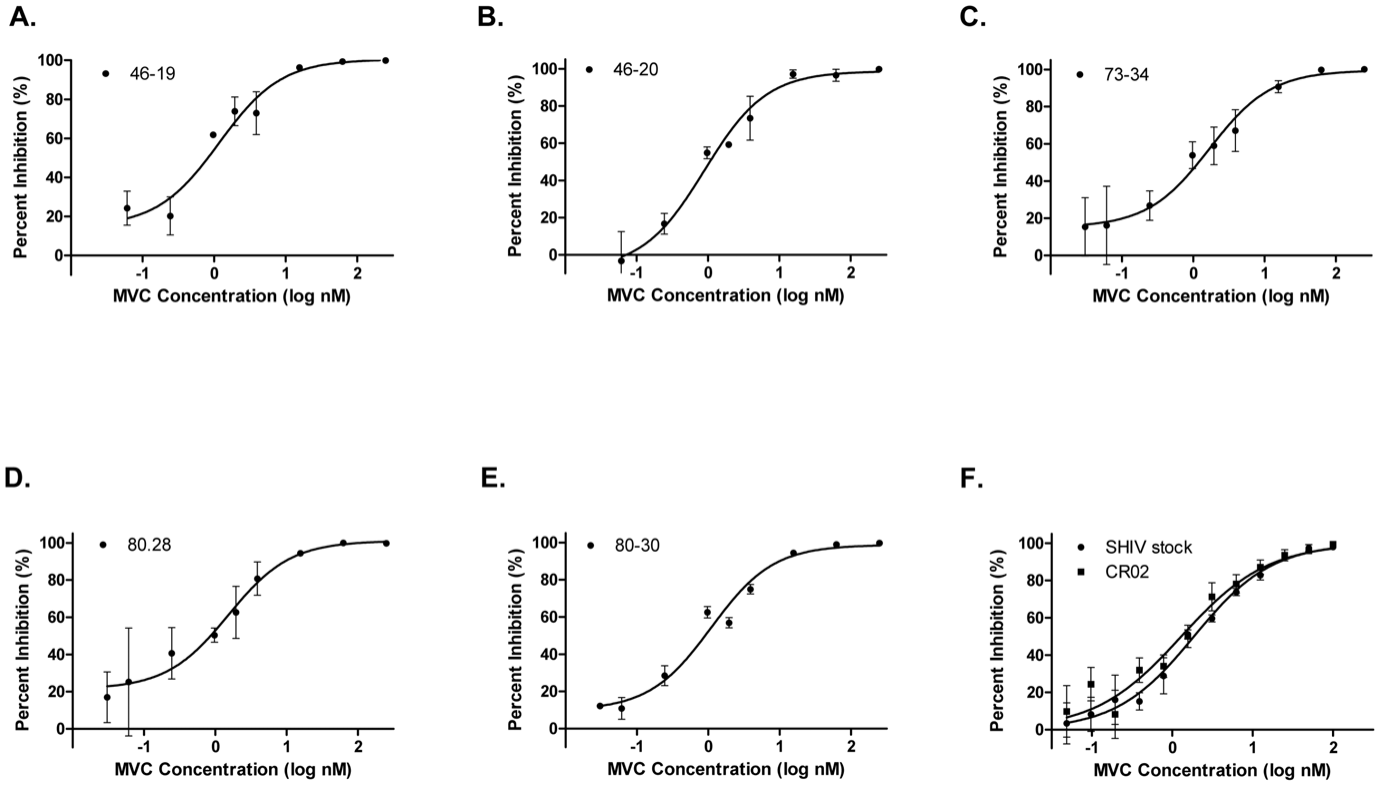
Maraviroc susceptibilities of pseudoviruses with full-length T1 envelopes. In each graph, the percentages of inhibition relative to the extent of virus replication in the no-MVC control at various MVC concentrations are shown. The MVC susceptibilities of the following clones are shown: (A) Mac46–19, the most prevalent Mac46 day 14 env clone, (B) Mac463–20, a minority day 14 Mac46 env clone, (C) Mac73–34, the most prevalent day 21 Mac73 env clone, (D) Mac803–28, the most prevalent day 14 Mac80 env clone, (E) Mac803–30, a minority day 14 Mac80 env clone, (F) SHIV stock and CR02, the most prevalent env clones from the pre-infection SHIV-162P3 stock and day 14 control macaque CR02, respectively. Error bars represent the standard errors of the means of results from at least two experiments, each performed in triplicate. Nonlinear regression with a variable slope was used to estimate a fitted curve. MVC, maraviroc.

To determine MVC susceptibility, we generated pseudoviruses derived from the most prevalent full-length *env* clone present in T_1_ plasma from Mac46, Mac73, Mac80, and CR02 [30], [31]. Two additional clones from animals Mac46 and Mac80 that contained sporadic gp160 mutations were also tested. Pseudoviruses that incorporated population-derived *env* from SHIV-162P3 stock and T_1_ CR02 were used to determine comparator MVC susceptibilities. All the *env*-pseudotype viruses were fully inhibited by MVC, with maximal percent inhibition (MPI) values of 99–100% (**Fig. 4**). The observed IC_50_ values for the SHIV-162P3 stock and T_1_ CR02 control virus were 1.87 nM (95% confidence intervals [CI], 1.45–2.41) and 1.30 nM (0.94–1.80), respectively. Viruses that incorporated the dominant T_1_ Mac46, Mac73, and Mac 80 *env* had MVC IC_50_s of 1.18 nM (0.54–2.58), 1.69 nM (0.76–3.76), and 1.53 nM (0.56–4.2), respectively. The minority Mac46-20 and Mac80-30 clones demonstrated MVC IC_50_s of 0.89 nM (0.49–1.61) and 1.10 nM (0.71–1.69). No statistically significant differences in MVC IC_50_s or MPIs were observed between any tested viruses. Hence SHIV infection was established in each macaque by CCR5-using virus; MVC susceptibilities did not differ from the infecting SHIV stock or a placebo-treated animal.

**Figure 5:**
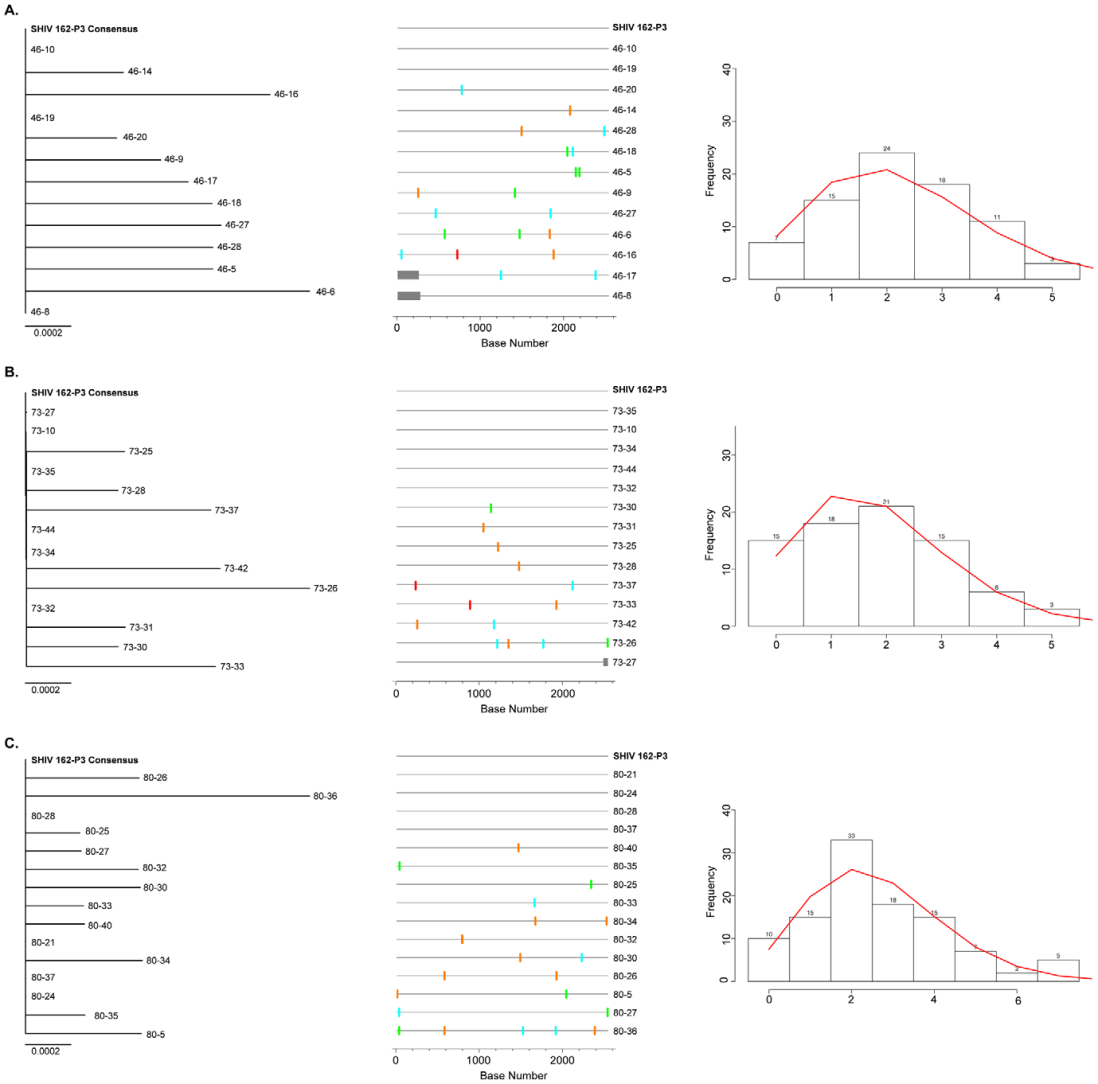
Neighbor-joining trees, Highlighter plots, and Hamming Distance frequency plots for independent T_1_ *env* clones. (A) Mac46, (B) Mac73, (C) Mac80. Only near full-length sequences were used to construct NJ trees; fewer sequences are included than in Figure 1. Highlighter plots indicate nucleotide variation from the consensus; a base mixture at a given position in the consensus sequence is indicated as a variation in the highlighter plot, even if that base is present in the consensus mixture. The horizontal scale bars represent genetic distance. Adenine, green; Cytosine, aqua; Thymine, red; Guanine, orange. Grey bars indicate missing sequence. Hamming Distance (HD) analyses removed APOBEC-modified positions from all sequences; theoretical pairwise HD frequency counts are shown as a continuous red line. Numbers above histogram columns denote the frequency observed for each HD.

We next investigated the effect of a MVC vaginal gel on the number of SHIV viruses that established the initial infections (**Fig. 5**) [32]. The phylogenetic estimates, supported by highlighter plot analyses, suggest that SHIV-162P3 infections in Mac46, Mac73, and Mac80 were established by a single, or a few very closely related, viral genotype(s). We observed the expected Poisson distribution of pairwise Hamming Distance frequency counts, consistent with transmission of a single viral variant in each animal [33].

**Figure 6:**
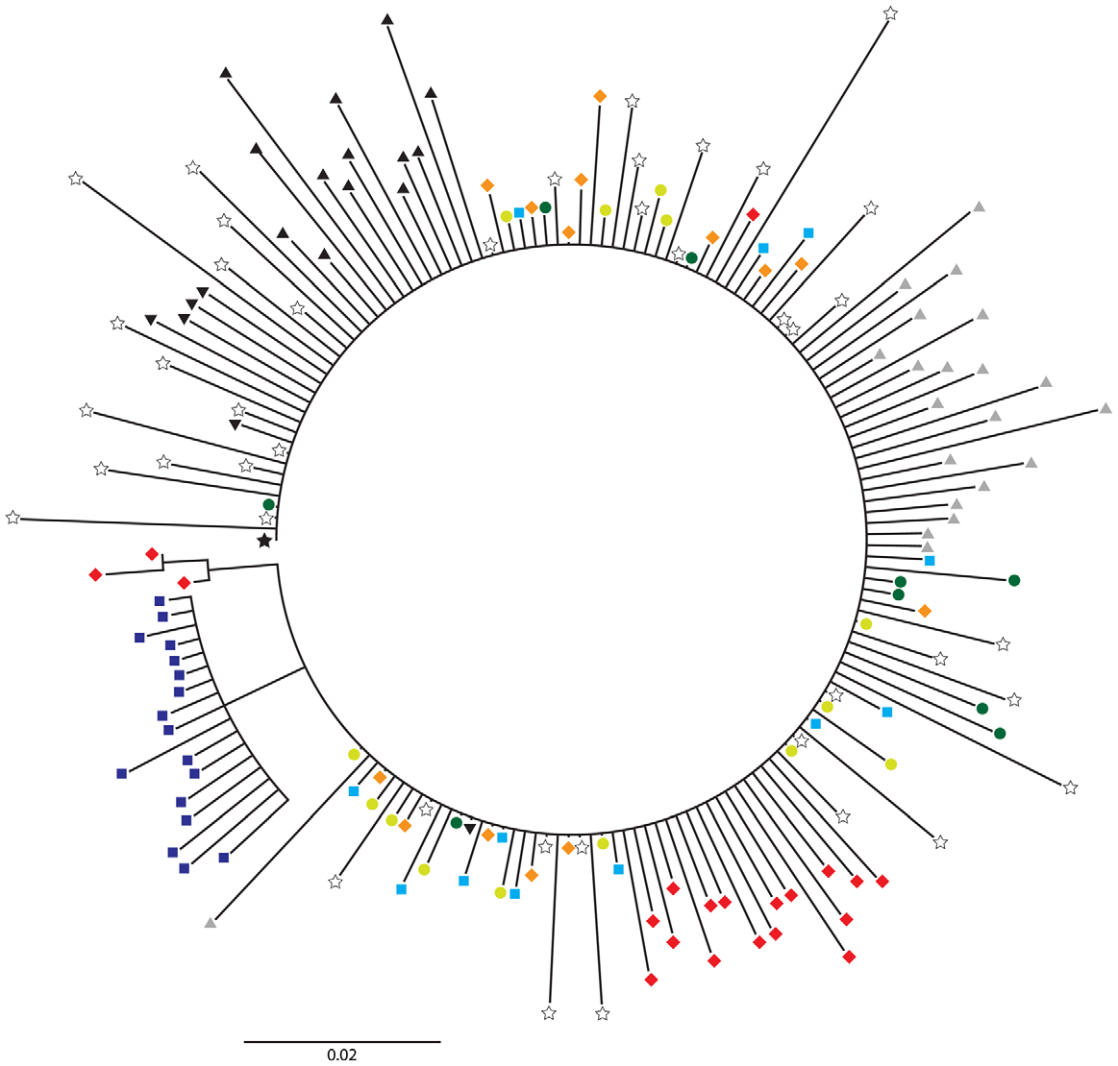
Phylogenetic analysis of T_1_ and T_2_ gp160 *env* sequences. Composite tree of 169 complete gp160 sequences that includes independent sequences isolated from the infecting SHIV-162P3 challenge stock and MVC-exposed and control macaques. Black star, SHIV-162P3 consensus *env* sequence; open stars, clonal SHIV-162P3 isolates; orange diamonds, day 14 Mac80; light green circles, day 21 Mac73; light blue squares, day 14 Mac46; red diamonds, day 70 Mac80; dark green circles, day 42 Mac73; dark blue squares, day 56 Mac46; grey triangles, day 14 control macaque CR02; black triangles, day 42 CR02; inverted black triangles, day 42 control macaque L375. Numerals indicate posterior probabilities of node support. The horizontal scale bar represents genetic distance.

Although the MVC gel did not affect the number of founder viruses or their susceptibility to MVC, it remained possible that it could have selected for particular transmitted lineages of the SHIV-162P3 stock. To evaluate the genetic relatedness of the infecting viruses in Mac46, Mac73 and Mac80, we constructed a Neighbor Joining tree, rooted on the SHIV-162P3 consensus sequence, that included full-length *env* sequences isolated at T_2_ (day 42) from the two control macaques, L375 and CR02, T_1_ sequences from CR02, and the SHIV-162P3 stock clones (**Fig. 6**). T_1_ sequences from Mac46, Mac73, and Mac80 shared a monophyletic cluster with clonal sequences from the SHIV-162P3 challenge stock, macaques CR02 and L375, and the derived SHIV consensus sequence. Some T_2_ *env* sequences diverged from those obtained at T_1_. T_2_ sequences from Mac46 and Mac80 each formed distinct clusters that did not mix with ones from other macaques; a subset of Mac80 T_2_ sequences were placed in the shared monophyletic lineage. Evolution of Mac73 T_2_ sequences from T_1_ sequences was not observed. Overall, the evolutionary relationships between the SHIV-162P3 stock and *env* sequences isolated over 70 days of observation did not appreciably differ between the MVC-exposed and control macaques.

## Discussion

We investigated the genotypic and phenotypic properties of SHIV-162P3 macaque infection in the presence of a MVC-containing vaginal gel and found that infecting viral populations closely resembled the stock inoculum. The above analyses imply that the presence of an MVC-based vaginal microbicide gel did not drive the selective transmission of MVC-insensitive variants to SHIV-162P3-challenged macaques. That is an important finding, given the uncertainty that surrounds the identification of a transmitted SHIV-162P3 variant with apparent resistance to vaginally administered PSC-RANTES [20], [21]. The infections in the three MVC recipients and the control animals were established by a similarly limited number of variants, just as was seen in untreated macaques challenged intra-vaginally with SIVmac251, and in sexually infected women [25], [34]–[39]. This finding is particularly noteworthy given that the animals in this and another recent study were treated with Depo-Provera to thin the vaginal epithelium and facilitate vaginal transmission [25]. The small number of HIV-1 variants that established infection in women using a tenofovir-containing vaginal microbicide was also not significantly different from control [40].

The relatively small number of sequences used to determine founder virus lineage is a limitation of this analysis. Although 13–15 SIV clones were sequenced from each macaque, there is at best a 70–80% probability by Poisson calculations that we did not miss a lineage present at 10% or less of the total transmitted viral population. Reassuringly, the conclusions of founder virus analyses have been confirmed when re-calculated with more comprehensive deep sequencing data sets [41], [42]. Within these limitations, we found no evidence that a MVC-based microbicide gel affects the types of genetic variants that ultimately establish infection, and we also showed that infections in vaginally-challenged macaques are established by one or a very few variants. Given the small number of infected animals we had available for study, animal and human participants in future trials of microbicide candidates should be studied to assess the generalizability of our findings.

An additional caveat is that the quasispecies diversity of a SHIV-162P3 challenge stock may not adequately mimic the diversity involved in sexual exposure to HIV-1. Intra-host HIV-1 envelope and V3 loop sequence diversity can range from 4–10%, a factor of 10 greater than the genetic distances observed in infecting SHIV-162P3 inocula [43], [44]. Under these circumstances, there could be subtle selective effects of a microbicide (MVC or other) on the infecting viral phenotype that we would not have detected in the present study. As an extreme example, a CCR5 inhibitor like MVC would inevitably not interfere with the transmissibility of CXCR4-using HIV-1 variants present in semen.

We could not evaluate whether MVC-resistant SHIV variants were locally selected under drug pressure and remained compartmentalized in the lower female genital tract. Pharmacokinetic studies have shown that plasma concentrations of MVC peak 2 hours after intravaginal administration of a 6 mM MVC-containing HEC gel at approximately 4 nM, a concentration at least twice that of the observed IC_50_s of viral isolates from this study [45]. MVC levels quickly decay thereafter and are undetectable in plasma 24 hours after gel administration. Plasma viremia is not detectable until days later, raising the possibility that the drug concentrations are not high or sustained enough outside the genital mucosa to select for viral resistance [10], [46].

We believe that the explanation for why some animals became infected despite the presence of a gel containing millimolar concentrations of MVC most likely is rooted in the pharmacology of inhibitor delivery (we note that 2 of the animals did receive sub-optimal MVC concentrations). Leakage of the gel and its active ingredient and/or a failure of the inhibitor to reach and occupy all the CCR5 targets in the lower female genital tract are probably responsible. While no protection will ever be 100% effective, the value of vaginal microbicides to prevent HIV-1 infection is now clear [9]. The development of better delivery systems and the use of ARV combinations can only increase the chances that a microbicide will eventually be of substantial value to populations of women at risk for heterosexual HIV-1 infection.

## Materials and Methods

### Ethics Statement

The Institutional Animal Care and Use Committee (IACUC) of Tulane University reviewed and approved all macaque procedures described (protocol permit number 3501). This study was carried out in strict accordance with the recommendations in the Guide for the Care and Use of Laboratory Animals of the National Institutes of Health (NIH) and with the recommendations of the Weatherall report, “The use of non-human primates in research”. All procedures were performed under anesthesia using ketamine, and all efforts were made to minimize suffering, improve housing conditions, and to provide enrichment opportunities (e.g., objects to manipulate in cage, varied food supplements, foraging and task-oriented feeding methods, interaction with caregivers and research staff).

### Macaque samples

We studied viral isolates from five rhesus macaques. Three animals, designated macaque 46 (Mac46), macaque 73 (Mac73), and macaque 80 (Mac80), received hydroxylethyl cellulose (HEC)-based vaginal gels containing 5.8 mM, 1.9 mM, and 0.6 mM of MVC, respectively, 30 min before an intra-vaginal challenge with 500 50% Tissue Culture Infectious Doses (TCID_50_) of SHIV162-P3, but became infected. Two infected control macaques, L375 and CR02, given an HEC placebo gel prior to SHIV-162P3 challenge, served as sources of comparator sequences. Two plasma samples collected from each macaque at an early time point (time 1, T_1_, days 14–21), when viral RNA was first detected, and a later time point (time 2, T_2_, days 42–70) after challenge were available for analysis.

### Cells and cell culture

TZM-bl and U87-CD4-CCR5 cells were obtained from the NIH AIDS Research and Reference Reagent Program (ARRRP). 293T cells were obtained from the American Type Culture Collection (ATCC, Manassas, VA). TZM-bl and 293T cell lines were maintained in Dulbecco modified Eagle medium with L-glutamine (DMEM, Gibco, Invitrogen) supplemented with 10% fetal bovine serum (Invitrogen), 100 U/ml penicillin and 100 µg/mL streptomycin (Cellgro, Mediatech) that was referred to as DMEM complete (DMEM-C). U87-CD4-CCR5 cells were grown in DMEM-C plus 300 µg/mL of geneticin (G418, Sigma) and 1 µg/mL puromycin (MP Biomedicals) to maintain CD4 and CCR5 coreceptor expression. All cell cultures were maintained at 37°C and 5% CO_2_.

### Maraviroc and susceptibility testing

Maraviroc was obtained from the NIH ARRRP. Clonal susceptibility testing was performed as previously described [28], [47].

### HIV-1 env cloning

Viral RNA was extracted from macaque plasma (QIAamp viral RNA mini kit, Qiagen) and *env* amplicons encoding gp160 were generated by nested PCR as described [28]. To minimize any potential founder effect, four independent RT reactions and PCR amplifications were performed and combined for each time point. These purified amplicons were then ligated into a TOPO- TA vector (Invitrogen) and electroporated into TOP10 cells. Subclones were isolated and sequenced by conventional (Sanger) methods as described [28]. Between 15 and 42 clones were sequenced per time point. All sequences were edited, aligned, and compiled with Geneious Pro version 5.4.6 [32]. Envelope sequences could not be amplified from L375 T_1_ plasma aliquots using multiple well-validated primer sets; this time point was excluded from further analyses.

### Sequence and Evolutionary analysis

The mean pairwise distance of 42 full-length env clones isolated from the SHIV-162P3 stock were calculated using MEGA5 [48]. The Shannon entropy at each position of the SHIV-162P3 stock inoculum aligned gp160 sequence data set were calculated with Entropy-One (www.hiv.lanl.gov/content/sequence/ENTROPY/entropy_one.html). Full-length *env* sequences were aligned, inspected with the Highlighter tool (http://www.hiv.lanl.gov/content/sequence/HIGHLIGHT/highlighter.html), and used to construct consensus neighbor-joining phylogenetic trees of the T_1_ sequences. Poisson-Fitter was used to calculate Pairwise Hamming Distance frequency counts (http://www.hiv.lanl.gov/content/sequence/POISSON_FITTER/poisson_fitter.html).

Full-length gp160 sequences isolated from the infecting SHIV-162P3 stock, control macaques L375 and CR02, and MVC vaginal gel-treated Mac46, Mac73, and Mac80 were included in phylogenetic analyses of *env* evolution. A FindModel analysis (www.hiv.lanl.gov/content/sequence/findmodel/findmodel.html) selected the Tamura-Nei model as the most appropriate for subsequent analyses [49]. Neighbor joining trees with a significance cutoff of 70% and the SHIV-162P3 challenge virus consensus *env* as an outgroup were constructed for gp160 sequences using Geneious; trees were resampled in 1,000 bootstrapped replicates [32]. An unrooted maximum likelihood tree of infecting clonal SHIV-162P3 sequences was generated with the PhyML plug-in of Geneious [50]. The ML tree was resampled in 1,000 bootstrapped replicates and optimized for tree topology and branch length.

### Virus construction

Recombinant virus that incorporated HIV-1 envelopes from Mac46, Mac73, Mac80, CR02 and the SHIV-162P3 inoculum were constructed by a modification of a previously described method [30], [31]. Briefly, the CMV promoter was amplified and attached to a 265 base-pair segment of *rev* from pNL43 using overlap PCR. A second overlap PCR was then performed to link CMV-rev to the cloned or uncloned env amplicon of interest. These CMV-rev-env amplicons were then co-transfected into 293T cells with an NL4-3 envelope-deleted vector [51].

To generate infectious virus, we transfected 293T cells with CMV-*rev*-*env* amplicons together with the *env*-deleted NL4-3. Briefly, 8×10^6^ 293T cells were plated and cultured overnight at 37°C. On the day of transfection, culture media was double washed with phosphate-buffered saline (PBS, Cellgro, Mediatech), replaced by fresh phenol red-negative DMEM-C and warmed to 37°C. We used the Fugene 6 protocol (Roche Molecular Biochemicals) to transfect cells as previously described [28], [47]. Supernatants were collected 48 hours later, passed through a 0.45 µM filter, centrifuged at 72,000 g for 90 min at 4°C, aliquoted and stored at −80°C until use. Viral titers were determined by endpoint dilution as described.

### Nucleotide sequence accession numbers

SHIV162-P3 *env* sequences were deposited in GenBank under accession numbers JN979788-JN979955. SGA SHIV162-P3 env sequences JN011407.1 - JN011423.1 were retrieved from GenBank on October 14, 2011.
